# Association of age-related cognitive and obstacle avoidance performances

**DOI:** 10.1038/s41598-021-91841-9

**Published:** 2021-06-15

**Authors:** Ryota Sakurai, Kentaro Kodama, Yu Ozawa, Frederico Pieruccini-Faria, Kimi Estela Kobayashi-Cuya, Susumu Ogawa

**Affiliations:** 1 Research Team for Social Participation and Community Health, Tokyo Metropolitan Institute of Gerontology, Tokyo, Japan; 2 University Education Center, Tokyo Metropolitan University, Tokyo, Japan; 3 Faculty of Sport Sciences, Waseda University, 2-579-15 Mikajima, Tokorozawa, Saitama 359-1192 Japan; 4 Division of Geriatric Medicine, Department of Medicine, University of Western Ontario, London, ON Canada; 5 Gait and Brain Lab, Parkwood Institute and Lawson Health Research Institute, London, ON Canada; 6 Center for Clinical Epidemiology and Health Technology Assessment, St. Luke’s International University, Tokyo, Japan

**Keywords:** Human behaviour, Cognitive ageing, Motor control

## Abstract

An association between cognitive impairment and tripping over obstacles during locomotion in older adults has been suggested. However, owing to its memory-guided movement, whether this is more pronounced in the trailing limb is poorly known. We examined age-related changes in stepping over, focusing on trailing limb movements, and their association with cognitive performance. Age-related changes in obstacle avoidance were examined by comparing the foot kinematics of 105 older and 103 younger adults when stepping over an obstacle. The difference in the clearance between the leading and trailing limbs (Δ clearance) was calculated to determine the degree of decrement in the clearance of the trailing limb. A cognitive test battery was used to evaluate cognitive function among older adults to assess their association with Δ clearance. Older adults showed a significantly lower clearance of the trailing limb than young adults, resulting in greater Δ clearance. Significant correlations were observed between greater Δ clearance and scores on the Montreal Cognitive Assessment and immediate recall of the Wechsler Memory Scale-Revised Logical Memory test. Therefore, memory functions may contribute to the control of trailing limb movements, which can secure a safety margin to avoid stumbling over an obstacle during obstacle avoidance locomotion.

## Introduction

Motor and sensory systems are linked by higher-order neurological processes and cognition, which are involved in planning movements and responding to environmental changes^1,2^. Recent studies have demonstrated that cognition plays an important role in regulating locomotion in older adults^1,3–5^, and the association between cognitive impairment and an increased risk of falls has thus been suggested^1,6^.

Tripping while stepping over an obstacle is one of the most common problems leading to falls, which can be affected by cognitive impairment^7,8^. Indeed, patients with Alzheimer’s disease (AD) have a higher frequency of contact with obstacles than healthy older adults^9^, and such contact seems to be more frequent in the trailing limb^10^. This may be because stepping over an obstacle with the trailing limb is a movement that is not visible, and thus, is guided by the working memory of the obstacle height^10–13^. This concept has also been confirmed in studies using quadrupedal animals, who cannot see their hindlimb movements, wherein AD model mice showed a higher frequency of contact of the hindlimbs during an obstacle avoidance task than non-AD model mice^14^. Hence, cognitive impairment, including age-related memory decline in humans, may contribute to a failed trailing limb movement during the stepping-over action, as indicated by the significantly lower foot clearance compared to that of the leading limb^15^. This assumption is reasonable considering that trailing limb movement during continuous obstacle avoidance is not visible in the peripheral visual field because of the human body structure. However, it remains unclear whether the trailing limb movement (i.e., low clearance) during obstacle avoidance is influenced by memory impairment due to aging.

There are discrepancies in previous findings regarding age-related changes in obstacle avoidance (i.e., presence or absence of age-related changes in obstacle avoidance behavior)^16,17^. Furthermore, the effects of age-related cognitive decline on the human trailing limb movements during obstacle avoidance are not well known. However, relevant human studies have implied that decline in foot clearance of the trailing limb is possibly affected by age-related changes, as observed in AD model mice. For instance, in a previous study with participants stepping over an obstacle with a lower height (10% of participants’ leg length), older adults showed slightly lower foot clearance of the trailing limb compared with young adults, whereas foot clearance of the leading limb did not change with age^18^. This resulted in foot clearance asymmetry, which suggests that the trailing limb movement may be affected by aging. Furthermore, older adults who were at a higher risk for falls, which was detected by their fall history and a decline in the lower extremity, showed lower trailing limb clearance, and therefore, higher foot clearance asymmetries than the low-risk older and young adults^19^. In this previous study, high-risk older adults with impairments of the lower extremity also showed a decline in cognitive functioning, suggesting that poor trailing limb movement could result from a lower cognitive performance.

Assuming that cognitive impairment affects foot clearance during obstacle avoidance, it may also suggest an influence on lower limb behavior during obstacle avoidance, including toe-obstacle distance and heel-obstacle distance. This is supported by a previous study that showed that patients with AD land their leading foot significantly closer to the obstacle than older controls^9^. Although the study suggests that age-related cognitive decline may affect the control of foot-obstacle distance for obstacle avoidance, no study to date has determined the cognitive correlates of such changes in limb control during obstacle avoidance in non-demented older adults.

Another important factor is the variability in each obstacle avoidance parameter (i.e., foot clearance, toe-obstacle distance, and heel-obstacle distance), which may be more related to cognitive functioning than to absolute values. Particularly, spatial variability is a measure of healthy cognitive control because it expresses corrective adjustments over lower limb movements^20,21^. Although the association between cognitive impairment and greater gait variability during obstacle negotiation has been suggested^22–24^, few reports highlight their association during a stepping-over action.

This study determined whether age-related changes in limb movements during obstacle avoidance arise from lower cognitive performance among older adults, focusing on the trailing limb movement. To this end, with foot clearance as the main endpoint, (1) comparisons were made between that of the leading and trailing limbs for young and older adults to determine an age-related decline in obstacle avoidance. Then, (2) the difference between these foot clearances (i.e., foot clearance asymmetry) was calculated to reveal the cognitive correlates of the impaired control of the trailing limb. In this case, we applied the difference value normalized by foot clearance of the leading limb to eliminate the possibility that the decrease in trailing limb clearance was simply due to sensorimotor function (e.g., muscle weakness). Additionally, (3) we examined age-related differences in parameters of the stepping-over action and their correlation with cognitive performance. We hypothesized a deterioration in the control of the trailing limb indicated by decreased foot clearance of the trailing limb compared to that of the leading limb and greater trial-to-trial variability of parameters of the stepping-over action will be observed among older adults. Furthermore, we hypothesized that its decrement would be associated with worse cognitive functioning, particularly memory decline. The findings of the present study can elucidate the role of cognition in lower limb control during obstacle avoidance and may help prevent tripping over an obstacle, which results in falls among older adults.

## Results

As a result of the obstacle avoidance task, four older and four young adults were excluded from the analysis because their data could not be parsed (e.g., missing due to technical issues). Thus, 103 young and 105 older adults were included in the analyses. Table 1 shows the participants’ characteristics. Almost one-third of our older participants (29.0%) were young older adults (< 75 years old). Due to the inclusion criteria, older participants naturally showed normal global cognitive function (mean MMSE = 28.5) and good mobility (mean usual gait speed = 1.36 m/s) for their age.

**Table 1:** Participant characteristics.

Variables	Young adults n = 103	Older adults n = 105
Age, mean (SD)	27.4 (7.5)	78.2 (5.6)
Female, n (%)	55 (53.4)	86 (81.9)
Lower limb length, cm, mean (SD)	81.0 (4.9)	76.0 5.4
Gait speed, m/s, mean (SD)		1.36 (0.24)
MMSE (/30), mean (SD)		28.5 (1.7)
MoCA (/30), mean (SD)		25.6 (3.4)
TMT-A, s, mean (SD)		41.4 (55.1)
TMT-B, s, mean (SD)		116.6 (45.0)
LM: immediate (/50), mean (SD)		19.1 (7.3)
LM: delayed (/50), mean (SD)		13.9 (7.8)

*MMSE* Mini-Mental State Examination, *MoCA* Montreal Cognitive Assessment, *TMT* Trail Making Test, *LM* Logical Memory subtest of the Wechsler Memory Scale.

### Aging effects on obstacle avoidance parameters

**Figure 1:**
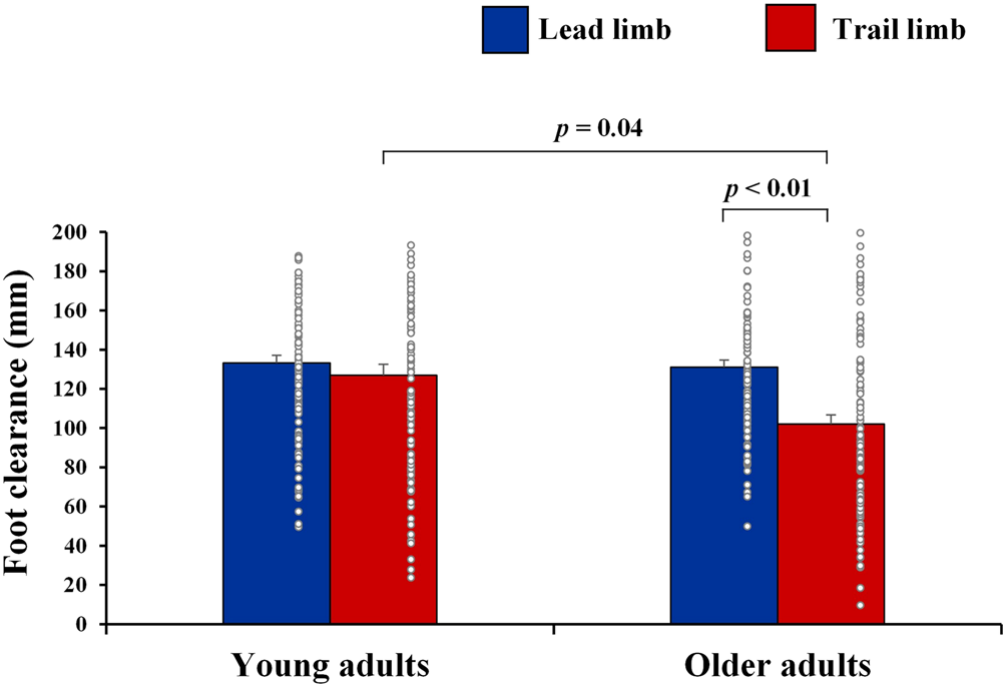
Comparisons of the leading and trailing limb clearances between young and older adults. Each comparison was adjusted for gender and the length of the lower limb. Bars and error bars indicate non-adjusted mean values and standard errors. Each plot indicates individual data points.

Figure 1 shows the results of the leading and trailing limb clearances among young and older adults. The repeated-measures ANOVA revealed no significant effects for the limb (*F*_1, 204_ = 1.2, *p* = 0.27, partial *η*^2^ = 0.01) or age group (*F*_1, 204_ = 0.7, *p* = 0.38, partial *η*^2^ = 0.002) factors; however, a significant interaction between the two factors was observed (*F*_1, 204_ = 8.6, *p* < 0.01, partial *η*^2^ = 0.03). The results of post-hoc analyses confirmed that there were significant differences in the clearance between the leading limb and trailing limb among older adults (*p* < 0.01), and in the clearance of the trailing limb between the young and older adults (*p* = 0.04). Furthermore, older adults showed a significant low clearance in their trailing limb compared to that of their leading limb and the trailing limb of young adults.

Table 2 shows the differences in other parameters of obstacle avoidance between young and older adults. The ANCOVA revealed that the toe-obstacle distance among older adults was significantly larger than that among young adults (*F*_1, 204_ = 11.2, *p* < 0.01, partial *η*^2^ = 0.05), and the heel-obstacle distance among older adults was significantly smaller than that among young adults (*F*_1, 204_ = 99.5, *p* < 0.01, partial *η*^2^ = 0.33). Furthermore, a greater variability in the heel-obstacle distance, compared to young adults, was observed among older adults (*F*_1, 204_ = 21.9, *p* < 0.01, partial *η*^2^ = 0.10). There were no significant age-related differences in variabilities in the toe-obstacle distance, leading foot clearance, or trailing foot clearance.

**Table 2:** The differences in parameters of obstacle avoidance between young and older adults.

Variables, mean (SE)	Young adults n = 103	Older adults n = 105	*p*-value
Variability in the LL clearance (CoV, %)	9.1 (0.5)	9.8 (0.5)	0.44
Variability in the TL clearance (CoV, %)	17.9 (1.0)	18.0 (1.0)	0.58
HO distance (mm)	293.8 (7.2)	179.8 (6.0)	< 0.01
Variability in the HO distance (CoV, %)	11.1 (0.6)	17.2 (1.0)	< 0.01
TO distance (mm)	143.0 (4.9)	160.1 (4.5)	< 0.01
Variability in the TO distance (CoV, %)	18.9 (1.2)	16.0 (1.1)	0.06

*LL* Leading Limb, *TL* Trailing Limb, *HO* Heel-obstacle, *TO* Toe-obstacle

### The association of age-related cognitive changes with obstacle avoidance

**Figure 2:**
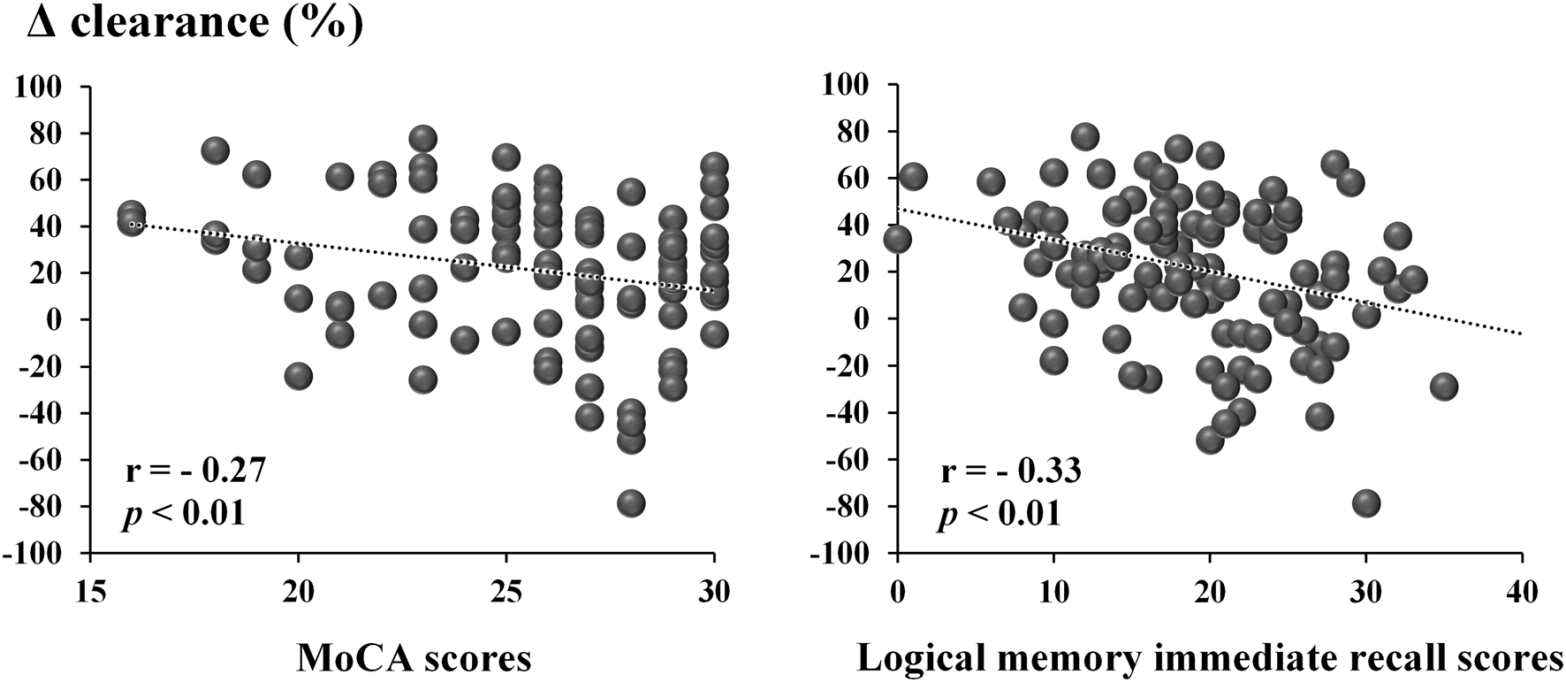
Scatter graphs of significant correlations between Δ clearance and scores on cognitive assessments. Δ clearance indicates the difference in clearance between the leading and trailing limbs calculated using the following formula: [(leading limb clearance − trailing limb clearance)/ leading limb clearance × 100]. The positive Δ clearance values represent a lower clearance of the trailing limb compared to that of the leading limb. Corrected covariates included gender, age, length of the lower limb, and gait speed. A Bonferroni correction of *p* < 0.01 was applied (*p* = 0.05/5, number of cognitive assessments).

Figure 2 shows the significant correlations between Δ clearance and the measurements of cognitive function among older adults. Partial correlation analyses adjusted for gender, age, the length of the lower limb, and gait speed showed that scores on the MoCA and LM immediate recall correlated negatively with Δ clearance, indicating that older adults who had lower cognitive scores showed a greater gap between leading and trailing limb clearances (i.e., decreased foot clearance of the trailing limb compared to that of the leading limb). In contrast, TMT-A (r = 0.08, *p* = 0.44), TMT-B (r = 0.17, *p* = 0.18), and LM delayed recall (r = 0.23, *p* = 0.02) did not correlate with Δ clearance.

Partial correlation analyses adjusted for the aforementioned covariates, which were performed on the basis of all resultant age-related significant differences between young and older adults, showed no significant correlations among the toe-obstacle distance, heel-obstacle distance, variability in the heel-obstacle distance, and cognitive variables (Table 3).

**Table 3:** Partial correlation coefficients among TO distance, HO distance, variability in the HO distance and cognition.

	MoCA	TMT-A	TMT-B	LM (immediate)	LM (delayed)
HO distance	− 0.01 (*p* = 0.94)	0.06 (*p* = 0.53)	− 0.03 (*p* = 0.79)	− 0.09 (*p* = 0.38)	− 0.15 (*p* = 0.14)
Variability in the HO distance	0.071 (*p* = 0.49)	− 0.004 (*p* = 0.97)	− 0.116 (*p* = 0.25)	0.083 (*p* = 0.41)	0.067 (*p* = 0.51)
TO distance	0.027 (*p* = 0.79)	0.022 (*p* = 0.83)	− 0.003 (*p* = 0.97)	0.084 (*p* = 0.41)	0.167 (*p* = 0.10)

*HO* Heel-obstacle, *TO* Toe-obstacle, *LM* Logical Memory subtest of the Wechsler Memory Scale. Partial correlation analyses adjusting for gender, age, length of the lower limb, and gait speed.

**Figure 3:**
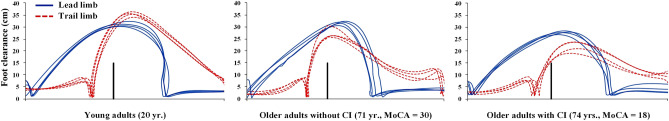
Typical toe trajectories in stepping over an obstacle among young and older adults with and without cognitive impairment (they were all women). *CI* cognitive impairment.

Figure 3 illustrates the typical toe trajectories in stepping over an obstacle among young adults, older adults without cognitive impairment (MMSE = 30; MoCA = 30; LM immediate recall = 26; LM delayed recall = 23), and older adults with cognitive impairment (MMSE = 27; MoCA = 18; LM immediate recall = 14; LM delayed recall = 9). Supporting the aforementioned results, the toe trajectory among older adults with cognitive impairment showed a lower clearance of the trailing limb compared to cognitively healthy controls (young and older adults without cognitive impairment), whereas no significant differences were found in the toe-obstacle distance and heel-obstacle distance between older adults with and without cognitive impairment.

## Discussion

We aimed to ascertain whether age-related cognitive decline resulted in a lower obstacle clearance of the trailing limb compared to that of the leading limb since its movement is considered to be regulated by memory. In this study, we observed a significantly lower clearance of the trailing limb among older adults than younger adults and, more importantly, a significant correlation was observed between cognitive decline and greater Δ clearance (i.e., lower trailing limb clearance compared to leading limb clearance). The results lend support to previous findings indicating that safe trailing limb movement (i.e., higher trailing limb clearance) during obstacle avoidance relies on memory of the obstacle’s height and position since it is not visible after the leading limb crosses the obstacle^10–13^.

In the present study, the clearance of the trailing limb was significantly lower among older adults than among young adults, whereas the leading limb was not significantly different among groups. Also, a lower clearance of the trailing limb compared to that of the leading limb was evident in those who had lower cognitive performance in terms of memory and global cognition as detected by the LM test and MoCA (Figs. 2 and 3). Scores on LM immediate and delayed recalls reflect the ability of short- and long-term episodic memory, and they are associated with the ability of working memory^25^. Previous studies on humans have implied that memory functions are involved in the control of limb movements during obstacle avoidance locomotion^23,26^, as well as among quadrupeds. For instance, accurate stepping movements of the trailing limb based on obstacle memory can be performed after a delay period of 2 minutes^27^. A lower clearance of the trailing limb compared to that of the leading limb was observed among older adults whose impaired memory function was assumed to diminish their ability to internally represent an obstacle encountered during walking^11^. This supports the concept that the memory of an obstacle encountered during walking would persist during obstacle avoidance^11–13,27^.

The MoCA correlated with Δ clearance in the present study. The MoCA examines important cognitive domains such as memory, executive functions, visuospatial abilities, language, attention, concentration, and temporal and spatial orientation, and has thus been used as a screening test for mild cognitive impairment (MCI)^28^. Our results raised the possibility that impairments in a wide range of cognitive functions (i.e., MCI), as well as memory decline, may be involved in the persistent internal representation of an obstacle during obstacle avoidance. Further studies are needed to confirm our results in MCI cohorts.

Possible brain regions responsible for persistent memory of an obstacle for controlling trailing limb movements during obstacle avoidance have been suggested in previous studies using quadruped animals such as cats, and the area 5 of the posterior parietal cortex (PPC) appears to be involved in this memory-guided movement^29–31^. McVea et al. proposed a conceptual model for the memory-guided limb movements, stating that an efference copy signal related to motor commands producing a stepping action in the leading limb (foreleg in the case of animals) initiates activity in the neurons in area 5, leading to the maintenance of the memory of the obstacle height^31^. This concept has been supported by findings that area 5 of the PPC deactivation, including lesions, resulted in the impairment of the ability of cats to maintain the height of an obstacle in their working memory and reduced clearance for both the leading and trailing hind leg steps^30,31^. Additionally, a significantly decreased perfusion in the PPC is observed among older adults with MCI who generally show lower scores in the MoCA and logical memory compared to cognitively healthy older adults^32^. Our results of the association between low clearance of the trailing limb compared to that of the leading limb and lower cognitive performance could be attributed to functional impairment of the PPC.

It has been shown that AD patients tend to land more closely to the obstacle after crossing it (i.e., shorter heel-obstacle distance) compared to cognitively healthy controls^9^, suggesting an increased risk of tripping on or collision with an obstacle. We also found that older adults were likely to land their leading foot closer to the obstacle after crossing it; however, the heel-obstacle distance among older adults was not associated with cognitive function, in contrast to previous findings. A possible interpretation of the discrepancy between the previous findings indicating the association of cognitive impairment with shorter heel-obstacle distance and those from the present study is that foot placement just before and after stepping over an obstacle is mainly affected by an impairment in the ability to control gait and posture, such as the neuromuscular system, rather than cognition.

Typical toe trajectories during the stepping-over action (Fig. 3) clearly showed a lower clearance of the trailing limb compared with that of the leading foot in older adults with cognitive impairment, which is consistent with the results that older adults who had lower cognitive scores showed a greater gap between leading and trailing limb clearances (i.e., Δ clearance). It is possible that the low clearance of the trailing limb, which was also observed in the example of the toe trajectory, could be attributed to a change in the toe trajectory due to placing their trailing foot further from the front of the obstacle, thus resulting in them landing close to the obstacle with their leading heel. However, this assumption is discounted because low and insignificant correlation coefficients among toe-obstacle distance, heel-obstacle distance, and clearance of the trailing limb were observed in both young and older adults (data not shown). Additionally, considering that the toe- and heel-obstacle distances were not associated with cognitive performance, the low clearance of the trailing limb, which is related to lower cognitive performance, may be independent of these behavioral changes before and after stepping over.

Positive values of Δ clearance in this study indicate a lower clearance of the trailing limb compared with that of the leading limb. Thus, our result showing the association between greater Δ clearance and lower score on the LM immediate recall implies that memory functions is related to the control of trailing limb movements, which can secure a safety margin to avoid stumbling over an obstacle during obstacle avoidance locomotion. However, caution should be exercised when interpreting the result because negative values of Δ clearance (i.e., higher trailing limb clearance compared to the leading limb) do not necessarily indicate that the trailing limb movement is based on memory for obstacles. Specifically, if the participants stepped over an obstacle based on good memory of that obstacle, they would show the same foot clearance of the trailing limb as that of the leading limb; moreover, the foot clearance of the trailing limb would not be higher than that of the leading limb. One possible reason for our result is that, from the perspective of self-protection, those who can maintain the memory of the obstacle height (i.e., have a good memory) may step over an obstacle conservatively to avoid tripping their trailing limb on an obstacle. Although previous findings that high-functioning older adults tend to conservatively estimate and perform their motor action support our speculation^33–36^, this remains to be determined in future studies.

Although we hypothesized that increased trial-to-trial variability in the stepping-over action would be associated with worse cognitive functioning, our results did not demonstrate a significant difference in foot clearance variabilities between young and older adults, and neither did a significant association between poor cognitive function and greater variability in foot clearances. This is consistent with a prior finding that AD patients did not show a significantly greater foot clearance variability in both the leading and trailing limbs compared with healthy controls, whereas increased gait variability during the approach phase was observed^22^. Therefore, it is believed that cognitive impairment may attenuate anticipatory gait adjustments during obstacle avoidance, resulting in greater gait variability when approaching an obstacle^23^, but no foot clearance during stepping over. Another possible reason for this lack of association can be explained in terms of the small number of trials for capturing the variabilities. Although these few trials were selected to avoid fatigue because our participants included older adults, four trials in our experiment might not have been sufficient to calculate and assess the variability in each obstacle avoidance parameter.

The strength of this study is that it is the first to show age-related lower clearance of the trailing limb among older adults compared to younger adults and the association between this lower clearance, compared to that of the leading limb, and poor cognitive function among older adults in a relatively large sample. However, there are limitations other than the possible issue of a small number of trials for capturing variabilities in each parameter, which should be considered when interpreting the results. The cross-sectional design of this study precludes us from exploring the causal relationship between the associations found. Although the present study used six cognitive assessments to capture participants’ cognitive profile, if we had measured other functional domains of cognition, it may have reinforced our findings regarding the association between Δ clearance and lower cognitive performance, particularly in memory. Our results were controlled for potential confounders; however, residual confounding covariates may still be present. Further longitudinal research should examine the causal relationship between lower cognitive performance and changes in obstacle avoidance, using other experimental settings.

In conclusion, our results showed that a lower clearance of the trailing limb compared with that of the leading limb was observed among older adults and was evident in those with lower cognitive performance. The findings of this study suggest that memory functions contribute to the control of limb movements, which can secure a safety margin to avoid stumbling on an obstacle during obstacle avoidance locomotion.

## Methods

### Participants

Older participants were volunteers recruited from a database of community-dwelling older adults available at the Tokyo Metropolitan Institute of Gerontology (TMIG). Participants were included in the study based on the following criteria: (1) ability to walk independently for 5 min; (2) being fully functional in instrumental activities of daily living (IADL) assessed using the TMIG Index of Competence, which is a questionnaire comprising three multidimensional subscales: IADL, intellectual activity, and social function^37^; (3) ability to complete both obstacle avoidance tasks and cognitive assessments. The exclusion criteria were as follows: (1) Parkinsonism or any other neurological disorder (e.g., severe stroke) with a residual motor deficit; (2) active osteoarthritis affecting lower limb performance; (3) dementia, which was determined by self-reported medical history and medical interview conducted by a specialist, or significant cognitive impairment detected by the cut-off of 24 points on the Mini Mental State Exam (MMSE)^38^, which has a maximum score of 30 points, with higher scores indicating higher overall cognitive functioning. We also confirmed that none of the participants wore multifocal spectacles that might lead them to misperceive objects. Younger participants were recruited from several universities as controls. We confirmed that they had no physical, neurological, or mental disorders and used no medication. In total, 109 older adults aged 78.1 ± 5.6 years, and 107 young adults aged 27.0 ± 5.8 years, participated in the study.

The Tokyo Metropolitan Institute of Gerontology Ethics Board approved this study. Informed consent was obtained from the participants during enrollment. The study was conducted in accordance with the principles of the Declaration of Helsinki.

### Measurements

Data on participants’ health conditions were obtained through interviews conducted before the obstacle avoidance task and cognitive assessments. The interview items included demographics, comorbidities, history of hospitalization, and medication use. The order of obstacle avoidance task trials and cognitive assessments were conducted randomly to prevent a potential order effect on motor and cognitive performance.

### Obstacle avoidance task

#### Experimental setup and apparatus

The experiment was conducted in a sound-isolated flat room illuminated with homogeneous white light. A rectangular solid obstacle made of white expanded polystyrene measuring 150 mm × 600 mm × 10 mm (height × width × depth), with L-brackets attached to the bottom to hold the obstacle upright. The color of the floor was dark gray to highlight the obstacle.

Foot kinematics data were collected using a three-dimensional motion capture system (OptiTrack V120: Trio, NaturalPoint, Inc.). It was located diagonally on the right side in front of the participant, 160 cm high, using a tripod and had a sampling frequency of 120 Hz. Reflective markers (9.5 mm diameter) were attached directly to the flat walking shoes prepared by the experimenter to estimate the toe and heel position: first and fifth toes and center of the heel on both sides of the feet. Markers were also placed on the upper front edge of the obstacle to determine its location and height within the motion capture system. Before the experiment, we confirmed that the participants’ movements when stepping over an obstacle could be captured with a camera without any problems. Data were collected using Motive software (NaturalPoint, Inc.) and analyzed using MATLAB (Mathworks, Sherborn, MA, USA). Time series data of each marker were smoothed by a second-order Butterworth low-pass filter with a 10 Hz cutoff frequency.

### Procedure

The obstacle was placed 150 cm from the participant. Then, on a verbal command of “go,” participants walked down the pathway at a self-selected pace and stepped over the obstacle in four steps. In this case, participants were instructed to start walking from their left foot, take (walk) three steps, place the next step over the obstacle from the right foot as the fourth step (i.e., left foot was the trailing foot), and keep walking until they reached the end line. No time restrictions were imposed on their performance. Several practice trials were conducted before the main trials until the stepping-over action became stable. After the practice trials, the participants were allowed to amend their starting position regarding the distance and back and forth to adjust their steps for a smooth stepping-over action. The experiment was performed four consecutive times.

**Figure 4:**
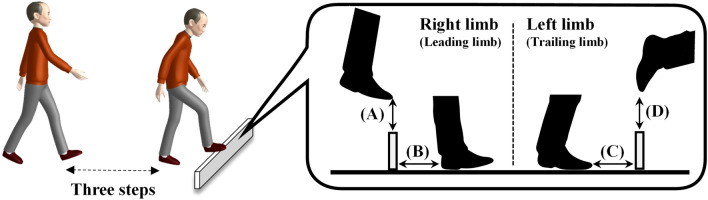
Schematic illustration of obstacle avoidance task and parameters. (**A**) Leading limb clearance; (**B**) Heel-obstacle distance; (**C**) Toe-obstacle distance; (**D**) Trailing limb clearance.

We measured the following variables as parameters of the stepping-over action (Fig. 4): (a) leading foot clearance, which is the vertical distance between the toe-tips detected by reflective markers attached on the first and little fifth toes of the leading limb (first limb to pass over the obstacle) and the upper edge of the obstacle as each respective marker passed over the obstacle; (b) trailing foot clearance, which is the vertical distance between the toe-tips of the trailing limb (the second limb to pass over the obstacle) and the top of the obstacle as it passed over the obstacle; (c) toe-obstacle distance, which is the horizontal distance between the trailing-limb toe tip and the obstacle right before stepping over the obstacle; (d) heel-obstacle distance, the horizontal distance between the heel tip of the leading limb and the obstacle for foot placement immediately after crossing the obstacle. The mean and variability (coefficient of variation, CoV, %) of each variable were calculated for the four trials.

### Cognitive assessments

To better understand the cognitive influence on obstacle avoidance, cognitive domains, namely global cognition, executive function, and memory, were evaluated using the Montreal Cognitive Assessment (MoCA), Trail Making Test (TMT)-A and-B, and the Logical Memory subtest of the Wechsler Memory Scale (LM), respectively. Clinical psychologists performed the assessments.

The MoCA comprises six domains examining the overall cognitive function: (i) time and place orientation, (ii) memory, (iii) visuospatial abilities, (iv) executive function, (v) attention and working memory, and (vi) language. It has a maximum score of 30-points, with higher scores indicating higher overall cognitive function^28^.

The TMT-A assesses simple visual search and motor speed skills^39^. Participants were asked to draw a line with a pencil to connect 25 printed numerals from 1 to 25 in an ascending order. The TMT-B assesses higher-order cognitive skills such as working memory and mental flexibility^39^. In this, participants performed a visual-motor task similar to the TMT-A, except this included connecting 13 numerical numbers and 12 Japanese hiragana characters while alternating numbers and letters in an ascending order. The shorter the time required for these tests, the higher the executive function.

For LM test, which comprehensively examines memory, participants were orally presented two short stories separately, and were then asked to recall each story verbatim (immediate recall)^40^. The maximum score for each story recall is 25 points (i.e., a total of 50 points). Approximately 30 min after the immediate recall, a free recall of the story was again elicited (delayed recall). Delayed recall tasks have the same scores (a total of 50 points).

### Covariates

Gender and the length of the lower limb (i.e., distance from the greater trochanter to the ground through the lateral malleolus) were adopted as covariates when examining age-related differences in the parameters of obstacle avoidance. In the case of examining the association of parameters of obstacle avoidance with cognition, gender, age, the length of the lower limb, and gait speed as a functional measure of the lower extremity were adopted as covariates. For gait speed, which was introduced to eliminate the confounding of the lower extremity dysfunction, a trained tester asked the participants to walk once along an 11-m straight walkway on a flat surface at their usual pace, and then to walk twice along the walkway at the fastest and safest pace possible. Speed was calculated at a steady state by including only 5 m of the center of the 11-m pathway. The first and last 3-m were considered as the acceleration and deceleration phases and were not in the speed calculation.

### Statistical analysis

The participants’ characteristics were summarized using mean and standard deviation (SD) or frequencies and percentages, as appropriate. To examine the difference in foot clearances of leading and trailing limbs between young and older adults, repeated-measures ANOVA with two independent factors, that is, limb (leading and trailing) and age group (young and older adults), was performed after adjusting for gender and the length of the lower limb. Furthermore, an ANCOVA adjusted for gender and the length of the lower limb, including other parameters such as the toe-obstacle distance, heel-obstacle distance, and variabilities in each parameter, was performed to compare the young and older adults and reveal age-related behavioral differences in obstacle avoidance, except the foot clearance.

To determine the degree of decrement in the clearance of the trailing limb compared with that of the leading limb, the difference in the clearance between the leading and trailing limbs (Δ clearance) was calculated using the following formula: [(leading limb clearance − trailing limb clearance)/ leading limb clearance × 100], referring to a previous study^19^. As mentioned in the Introduction section, the difference value was normalized by leading foot clearance to eliminate the possibility that the decreased trailing limb clearance was simply due to muscle weakness. The positive Δ clearance values represent a lower trailing limb clearance compared to that of the leading foot, while zero Δ clearance values indicate that the obstacle clearance of the leading and trailing limbs is the same height. Due to the observed multicollinearities among cognitive measures, we performed partial correlation analyses (adjusting for gender, age, length of the lower limb, and gait speed) for each cognitive factor to examine their associations with Δ clearance among older adults. Similarly, partial correlation analyses adjusting for the aforementioned covariates were performed for older adults to examine respective associations among parameters of obstacle avoidance other than foot clearances and cognitive variables on the basis of all resultant significant age-related differences between young and older adults.

All statistical analyses were performed using IBM SPSS Statistics, Version 20.0 (SPSS Inc., Chicago, IL, USA). Statistical significance was set at *p*-value less than 0.05. To avoid type 1 error, a Bonferroni correction of *p* < 0.01 was applied in each correlation analysis (*p* = 0.05/5, number of cognitive assessments).

## Data Availability

The datasets analyzed during this study are available from the corresponding author upon reasonable request and after approval by the institutional authorities.
